# Incidence, mortality, and survival of hematological malignancies in Northern Italian patients: an update to 2020

**DOI:** 10.3389/fonc.2023.1182971

**Published:** 2023-07-18

**Authors:** Lucia Mangone, Domenico Penna, Francesco Marinelli, Francesca Roncaglia, Isabella Bisceglia, Francesco Merli, Alessia Ruffini, Barbara Gamberi, Alessia Tieghi, Riccardo Valli, Laura Albertazzi, Mauro Iori, Paolo Giorgi Rossi, Claudia Vener, Fortunato Morabito, Antonino Neri, Stefano Luminari

**Affiliations:** ^1^ Epidemiology Unit, Azienda USL-IRCCS di Reggio Emilia, Reggio Emilia, Italy; ^2^ PhD Program in Clinical and Experimental Medicine, University of Modena and, Reggio Emilia, Italy; ^3^ Hematology Unit, Azienda USL- IRCCS di Reggio Emilia, Reggio Emilia, Italy; ^4^ Gruppo Amici Dell’Ematologia Foundation-GrADE, Reggio Emilia, Italy; ^5^ Pathology Unit, Azienda USL- IRCCS di Reggio Emilia, Reggio Emilia, Italy; ^6^ Laboratory of Clinical Chemistry, Azienda USL- IRCCS di Reggio Emilia, Reggio Emilia, Italy; ^7^ Medical Physics Unit, Azienda USL-IRCCS di Reggio Emilia, Reggio Emilia, Italy; ^8^ Department of Oncology and Hemato-oncology, Università di Milano, Milano, Italy; ^9^ Biotechnology Research Unit, AO di Cosenza, Cosenza, Italy; ^10^ Scientific Directorate, Azienda USL-IRCCS di Reggio Emilia, Reggio Emilia, Italy; ^11^ Chimomo Department, University of Modena and Reggio Emilia, Reggio Emilia, Italy

**Keywords:** hematological malignancies, incidence, mortality, survival, trends, cancer registry, digital health

## Abstract

**Background:**

Hematological malignancies (HMs) represent a heterogeneous group of diseases with diverse etiology, pathogenesis, and prognosis. HMs’ accurate registration by Cancer Registries (CRs) is hampered by the progressive de-hospitalization of patients and the transition to molecular rather than microscopic diagnosis.

**Material and methods:**

A dedicated software capable of automatically identifying suspected HMs cases by combining several databases was adopted by Reggio Emilia Province CR (RE-CR). Besides pathological reports, hospital discharge archives, and mortality records, RE-CR retrieved information from general and biomolecular laboratories. Incidence, mortality, and 5-year relative survival (RS) reported according to age, sex, and 4 HMs’ main categories, were noted.

**Results:**

Overall, 7,578 HM cases were diagnosed from 1996 to 2020 by RE-CR. HMs were more common in males and older patients, except for Hodgkin Lymphoma and Follicular Lymphoma (FL). Incidence showed a significant increase for FL (annual percent change (APC)=3.0), Myeloproliferative Neoplasms (MPN) in the first period (APC=6.0) followed by a significant decrease (APC=-7.4), and Myelodysplastic Syndromes (APC=16.4) only in the first period. Over the years, a significant increase was observed in 5-year RS for Hodgkin -, Marginal Zone -, Follicular - and Diffuse Large B-cell-Lymphomas, MPN, and Acute Myeloid Leukemia. The availability of dedicated software made it possible to recover 80% of cases automatically: the remaining 20% required direct consultation of medical records.

**Conclusions:**

The study emphasizes that HM registration needs to collect information from multiple sources. The digitalization of CRs is necessary to increase their efficiency.

## 1 Introduction

Hematological malignancies (HMs) are a heterogeneous group of neoplasms, including distinctive entities with different etiology, pathogenesis, prognosis, and treatment (1). Various papers dealing with different aspects of HM epidemiology have been published (2–9), with the most systematic reports on incidence, survival, and prevalence coming from the US Surveillance Epidemiology and End Results (SEER) (10), the Cancer Registries (CR) in UK and Nordic countries (11–14). Registration of HMs is somewhat problematic (15) since the care of many of these diseases is not based anymore on hospitalization, particularly in the initial phases of the disease, and because their diagnosis is shifting increasingly to molecular tests instead of only microscopic assessment (16). Thus, CRs activity, traditionally based on the three characteristic data sources, i.e., pathology, hospital, and mortality records, may miss incident cases or register them several years after diagnosis. A resulting strong suggestion was to include new data sources among those commonly used to identify cases by CRs, such as molecular laboratory and drug prescription databases (17).

In Italy, the most updated national epidemiological data concerning HMs report incidence estimates for 2019 grouped into four major classes, namely Hodgkin Lymphoma (HL), non-Hodgkin Lymphoma (NHL), Plasma Cell Neoplasms (PCN), and Leukemia (13). The estimates are based on cases registered by CRs from 2010 to 2015. Although the regions of northern Italy had a higher incidence for almost all types of solid tumors, the national data concerning HMs were more homogenous, with the only exception being NHLs, which showed a higher incidence in Northern Italy. HM mortality was also comparatively homogenous, excluding NHLs that showed a higher rate in also in Northern Italy. Moreover, survival of the HMs four major groups mentioned above showed a marked improvement for patients diagnosed in 2005-2009 compared to patients diagnosed in 1990-1994 for both genders (18). Italian patients affected by HMs showed longer survival than the European average but shorter than what was reported in the US (14). Incidence trends were stable for HL, while they were decreasing for leukemia, myeloma, and NHL (for the latter, mainly in females) (19).

This study aimed to describe the incidence, mortality, and relative survival (RS) of patients diagnosed with HMs from 1996 to 2020 in a northern Italy province using a CR-dedicated software capable of automatically identifying suspected HMs cases by a robust digital health tool combining several databases.

## 2 Materials and methods

### 2.1 Data sources

HMs incidence data from 1996 to 2020 were obtained from the Reggio Emilia CR (RE-CR) (approved by the provincial Ethics Committee of Reggio Emilia, Protocol no. 2014/0019740, on August the 4th, 2014). HM cases were defined according to the International Classification of Diseases for Oncology, Third Edition (ICD-O-3) (20) and included ICD-O-3 codes. They were classified into 14 main categories, as reported in **Table 1**. Diagnostic groups were defined according to the main cell of origin. Groups with a small number of cases were merged into larger groups considering similarities in the pathology and biology of the disease. For this reason, Nodular lymphocyte-predominant Hodgkin lymphoma was lumped together with classical HL, and Plasma cell Leukemias were lumped with Multiple Myeloma. The remaining rare subtypes were merged and classified as “others”.

**Table 1:** Reggio Emilia Cancer Registry. Categories of hematological malignancies and respective morphological ICDO-3 codes.

Disease categories	Classification	Histotypes	ICD-O-3 morphologic code
**Lymphoid**	B-Cell/aggressive	Hodgkin Lymphoma (HL)	9650/3-9667/3
	B-Cell/indolent	Chronic Lymphocytic Leukemia/Small Lymphocytic Lymphoma (CLL/SLL)	9670/3, 9823/3
	B-Cell/indolent	Lymphoplasmacytic Lymphoma (LPL)	9671/3, 9761/3
	B-Cell/indolent	Marginal Zone Lymphoma (MZL)	9689/3, 9699/3
	B-Cell/indolent	Follicular Lymphoma (FL)	9690/3-9698/3
	B-Cell/Aggressive	Mantle Cell Lymphoma (MCL)	9673/3
	B-Cell/Aggressive	Diffuse Large B-Cell Lymphoma (DLBCL)	9675/3 9687/3
	T-Cell/indolent and aggressive	Mature T-cell and NK-cell neoplasms	9700/3, 9701/3,9702/3, 9705/3, 9709/3, 9714/3, 9708/3, 9717/3, 9716/3, 9718/3, 9719/3, 9827/3, 9834/3
	Plasma Cell	Plasma Cell Neoplasms (PCN)	9731/3, 9732/3, 9733/3, 9734/3
	B/T-cell/precursor	B or T Cell Acute Lymphoblastic Lymphoma/Leukemia (ALL)	9729/3, 9835/3, 9836/3, 9837/3
**Myeloid**	Myeloid/chronic	Myeloproliferative Neoplasms (MPN)	9863/3, 9875/3, 9876/3, 9945/3, 9950/3, 9960/3, 9961/3, 9962/3, 9963/3, 9964/3, 9975/3
	Myeloid/chronic	Myelodysplastic Syndromes (MDS)	9980/3- 9989/3
	Myeloid/acute	Acute Myeloid Leukemia (AML)	9053/3, 9800/3, 9801/3, 9820/3, 9840/3, 9861/3-9867/3, 9871/3-9874/3, 9891/3-9897/3, 9910/3, 9920/3, 9930/3
Others			9590/3, 9831/3, 9832/3

ICD-O, International Classification of Diseases; HL, Hodgkin Lymphoma; CLL/SLL, Chronic Lymphocytic Leukemia/Small Lymphocytic Lymphoma; LPL, Lymphoplasmacytic Lymphoma; MZL, Marginal Zone Lymphoma; FL, Follicular Lymphoma; MCL, antle Cell Lymphoma; DLBCL, Diffuse Large B-Cell Lymphoma; ALL, Acute Lymphoblastic Lymphoma/Leukemia; PCN, Plasma Cell Neoplasms; MPN, Myeloproliferative Neoplasms; MDS, Myelodysplastic Syndromes; AML, Acute Myeloid Leukemia.

Others include: 9590/3 (Malignant lymphoma NOS: 94 cases).

9831/3-9832/3 T cell granular lymphocytic leukemia, prolymphocytic leukemia (22 cases).

The primary information sources of the RE-CR were histopathological reports, hospital discharge records, and mortality data. The RE-CR covered a population of 531,891 inhabitants and was considered of high-quality data with 98% of histopathological confirmations (compared with the national median of 96%) and a low rate of Death Certificate Only (less than 0.1%, compared with the national 1%) (21, 22). Furthermore, RE-CR was the only Italian CR with published data on all tumors and their most frequent sites updated to 2020 (23).

### 2.2 Digital health tools

Since HMs have different pathogenesis, behavior, and classification compared to solid tumors, we have developed new tools to register these specific neoplasms. New information flows have been added to the traditional information sources and integrated with laboratory tests, diagnostic reports, and information from general practitioners. New sources of information for HMs that included laboratory data reporting immunophenotypic, cytogenetic, and genomic aberrations are mandatory to assure the completeness and accuracy of case records.

In particular, the RE-CR algorithm identifies suspected cases by combining the three traditional databases (pathological reports, hospital discharge archives, and mortality records) with general and biomolecular laboratory tests, according to a deterministic list of diagnoses. All suspected cases are accompanied by a morphological code that comes from a pathological laboratory (SNOMED and ICD-O-3) or, when not present, from discharge records (ICD-9-CM) or the cause of death (ICD-10). The presence of any issue (e.g., previous solid or hematological tumors, unclear residential history, etc.) is highlighted so that the registrar can manually check for possible errors. In recent years, the RE-CR has developed an algorithm to further improve the quality of the cases by matching the pathological reports with a library of specific diagnostic terms thus improving the specificity of the SNOMED code indicated by the pathologist. For each suspected case, the registrar has access to all the current information in the Local Health Authority Data Warehouse (LHADW) organized in order of relevance, by date, and mention of oncological disease. In addition to the primary information sources, the LHADW contains imaging tests, outpatient visits, letters of discharge, and therapies provided by the six hospitals of the Reggio Emilia province.

Furthermore, every case proposed by the algorithm was validated manually: the registrars can confirm the proposed topography and morphology, change the date of diagnosis, or other data. Moreover, the RE-CR algorithm lets us provide detailed information on HMs, directly comparing RE-CR epidemiological data with those from the US SEER program (10).

### 2.3 Statistical analyses

The units of analysis are new HMs (the principal ICD-O-3 codes were: C09, C16-18, C34, C38, C41-42, C44, C48, C77, C71, C80) occurring from 1996 to 2020, second malignancies after non-hematologic malignancies were included; second malignancies after a registered HM were excluded. Descriptive analyses by age, sex, 14 HMs main categories (**Table 1**), and calendar period of a cancer diagnosis are presented. For age groups, specific incidence and mortality rates were calculated using the Province of Reggio Emilia (recorded on January 1st of each year) as denominators. The direct method was applied to standardize incidence rates, using the 2013 European Standard Population as a reference. Relative survival estimates net survival without other causes of death. It is defined as the ratio of observed in a cohort of cancer patients to the proportion of expected survivors in a comparable set of cancer-free individuals. For each HM group, relative survival was estimated with the Pohar Perme method; with this estimator, net survival for a cohort is estimated by weighting by the inverse of the individual-specific expected survival probabilities. The weights inflate the observed person-time and number of deaths to account for person-time and deaths not observed because of mortality due to competing causes (24). To estimate the general population mortality, we used calendar year-specific life tables for the Province of Reggio Emilia, provided by ISTAT (25). Changes in RS across the four 5-year periods (from 1996 to 2015) were tested for trends using Poisson regression models with the period as the independent continuous variable and the number of predicted events from Pohar’s 5-year RS estimate for each period and the period-specific incident cases as exposed population. Analyses were performed using STATA 16.1 software. Trends over time were analyzed by calculating the annual percent change (APC) in age-standardized rates using Joinpoint Regression analysis (26).

## 3 Results

In the province of Reggio Emilia, the availability of dedicated software, introduced in 2012, made it possible to automatically record over 80% of the cases, while in the remaining 20%, it was necessary to retrieve the information by directly consulting the medical records. Overall, 7,578 HM incident cases were registered (4,116 in males and 3,462 in females), showing a higher incidence for males (male/female ratio = 1.2) with the only exception of Follicular Lymphoma (FL) more represented in women (male/female ratio = 0.9) (**Table 2**).

**Table 2:** The number of cases of hematological malignancies and incidence age-Standardized Rates (European Standard Population 2013) per 100,000 person-years in registered in the Province of Reggio Emilia between 1996 and 2020 by sex.

	Males		Females		All	Male: Female ratio	SR (95%CI)
	n (%)	SR (95%CI)	n (%)	SR (95%CI)	n (%)		
Hodgkin Lymphoma (HL)	282 (57.3)	3.9 (1.6-6.4)	210 (42.7)	4.5 (1.8-7.3)	492 (6.5)	1.3	4.2 (2.4-6.0)
Chronic Lymphocytic Leukemia/Small Lymphocytic Lymphoma (CLL/SLL)	495 (56.1)	2.7 (0.7-4.6)	388 (43.9)	2.2 (0.7-3.8)	883 (11.7)	1.3	2.4 (1.2-3.6)
Lymphoplasmacytic Lymphoma (LPL)	112 (58.3)	0.8 (0.1-1.6)	80 (41.7)	1.6 (0.2-2.9)	192 (2.5)	1.4	1.2 (0.3-2.0)
Marginal Zone Lymphoma (MZL)	184 (54.0)	2.0 (0.4-3.6)	157 (46.0)	1.0 (0.1-2.2)	341 (4.5)	1.2	1.4 (0.5-2.4)
Follicular Lymphoma (FL)	199 (47.8)	3.3 (1.1-5.6)	217 (52.2)	5.2 (2.7-7.7)	416 (5.5)	0.9	4.4 (2.7-6.1)
Diffuse Large B-Cell Lymphoma (DLBCL)	489 (52.2)	8.0 (4.6-11.5)	447 (47.8)	5.8 (3.0-8.7)	936 (12.3)	1.1	6.8 (4.6-9.2)
Mature T-cell and NK-cell neoplasms	180 (63.8)	1.0 (0.2-2.2)	102 (36.2)	2.8 (0.9-4.7)	282 (3.7)	1.8	1.9 (0.8-3.1)
Mantle Cell Lymphoma (MCL)	78 (74.3)	1.9 (0.2-3.5)	27 (25.7)	0.2 (0.1-0.6)	105 (1.4)	2.9	1.0 (0.2-1.8)
Plasma Cell Neoplasms (PCN)	581 (50.7)	8.5 (5.0-11.9)	565 (49.3)	7.4 (4.4-10.3)	1,146 (15.1)	1.0	7.8 (5.6-10.0)
Acute Lymphoblastic Lymphoma/leukemia (ALL)	118 (57.0)	3.1 (1.0-5.3)	89 (43.0)	0.8 (0.1-1.9)	207 (2.7)	1.3	1.9 (0.7-3.1)
Myeloproliferative Neoplasms (MPN)	588 (54.2)	8.6 (5.1-12.2)	496 (45.8)	5.5 (2.9-8.2)	1,084 (14.3)	1.2	6.9 (4.7-9.0)
Myelodysplastic Syndromes (MDS)	378 (53.3)	4.2 (1.8-6.6)	331 (46.7)	3.0 (1.2-4.9)	709 (9.4)	1.1	3.4 (2.0-4.9)
Acute Myeloid Leukemia (AML)	375 (56.0)	5.1 (2.4-7.8)	294 (44.0)	6.0 (3.3-8.6)	669 (8.8)	1.3	5.5 (3.6-7.3)
Others	57 (49.1)	0.4 (0.1-1.1)	59 (50.9)	0.6 (0.1-1.4)	116 (1.5)	1.0	0.5 (0.0-1.1)
**Total**	**4,116 (54.3)**		**3,462 (45.7)**		**7,578 (100)**	**1.2**	

SR, age-Standardized Rates (European Standard Population 2013).

Considering all cases, PCNs were the most frequent HMs (15.1% of all cases), followed by Myeloproliferative Neoplasms (including Polycythemia Vera 221 cases; Myelofibrosis 215 cases, Essential Thrombocythemia 209 cases; Chronic Myeloid Leukemia BCR/ABL negative, 122 cases; and Chronic Myeloid Leukemia BCR/ABL positive, 127 cases) (14.3%) and Diffuse Large B-Cell Lymphoma (DLBCL) (12.3%).

The probability of being diagnosed with an HM increased significantly with age (**Table 3**). In some groups of HMs, only a limited number of cases have been recorded in patients younger than 18 years. Conversely, in most cases, tumors affected patients >65 years, except for the HL and FL, which were more frequent in the age of 18-65 years (**Table 3**). In patients <18 years, in addition to the 34 cases of HL, we recorded 93 cases of precursor B or T lymphoblastic lymphoma/leukemia (45% of the ALL subgroup). The overall median age at HM diagnosis was 70 years, being 40 and 21 years in HL and ALL, respectively.

**Table 3:** The number of cases of hematological malignancies and incidence age-Standardized Rates (European Standard Population 2013) per 100,000 person-years registered in the Province of Reggio Emilia between 1996 and 2020 by age groups.

	<18 years		18-65 years		≥65 years		Age at diagnosis
	n (%)	SR (95%CI)	n (%)	SR (95%CI)	n (%)	SR (95%CI)	Median (IQR)
Hodgkin Lymphoma (HL)	34 (6.9)	0.4 (0.0-1.2)	358 (72.8)	5.8 (2.9-8.7)	100 (20.3)	0.8 (0.1-1.7	40 (35)
Chronic Lymphocytic Leukemia/Small Lymphocytic Lymphoma (CLL/SLL)	0 (0.0)	–	284 (32.2)	1.0 (0.1-2.2)	599 (67.8)	2.0 (0.8-3.2)	71 (17)
Lymphoplasmacytic Lymphoma (LPL)	0 (0.0)	–	55 (28.6)	1.1 (0.0-2.2)	137 (71.3)	0.9 (0.0-1.9)	71 (15)
Marginal Zone Lymphoma (MZL)	1 (0.3)	–	143 (41.9)	0.6 (0.0-1.5)	197 (57.8)	2.4 (0.6-4.2)	69 (21)
Follicular Lymphoma (FL)	3 (0.7)	–	222 (53.4)	2.8 (1.1-4.5)	191 (45.9)	2.8 (1.3-4.2)	63 (18)
Diffuse Large B-Cell Lymphoma (DLBCL)	11 (1.2)	.	359 (38.3)	3.7 (1.7-5.7)	566 (60.5)	4.1 (2.4-5.8)	70 (24.)
Mature T-cell and NK-cell neoplasms	2 (0.7)	–	137 (48.6)	2.0 (0.5-3.6)	143 (50.7)	0.9 (0.0-1.9)	65.5 (21)
Mantle Cell Lymphoma (MCL)	0 (0.0)	–	34 (32.4)	0.4 (0.0-1.1)	71 (67.6)	1.7 (0.2-3.2)	71 (17)
Plasma Cell Neoplasms (PCN)	0 (0.0)	–	330 (28.8)	3.5 (1.6-5.3)	816 (71.2)	5.2 (3.3-6.9)	73 (17)
Acute Lymphoblastic Lymphoma/leukemia (ALL)	93 (44.9)	1.3 (0.1-2.5)	79 (38.2)	0.7 (0.0-1.7)	35 (16.9)	1.1 (0.0-2.4)	21 (47)
Myeloproliferative Neoplasms (MPN)	3 (0.3)	–	457 (42.2)	3.7 (1.6-5.8)	624 (57.6)	6.2 (3.6-	68 (24)
Myelodysplastic Syndromes (MDS)	0 (0.0)	–	79 (11.1)	1.0 (0.0-2.2)	630 (88.9)	3.3 (1.7-4.9)	79 (13)
Acute Myeloid Leukemia (AML)	15 (2.2)	–	241 (36.0)	2.0 (0.6-3.4)	413 (61.7)	4.0 (2.4-5.7)	72 (24)
Others	0 (0.0)	–	39 (33.6)	0.4 (0.0-1.1)	77 (66.4)	0.6 (0.0-1.4)	72 (22.5)
**Total**	**162 (2.1)**		**2,817 (37.2)**		**4,599 (60.7)**		**70 (2)**

SR, age-Standardized Rates (European Standard Population 2013); IQR, Interquartile range.

**Table 4** shows the numbers and age-standardized incidence rates of HMs by quinquennium over the 25 years of the study. An increase in incidence is appreciable for FL, MPN, and MDS groups. However, it cannot be excluded that the low number of cases in the 1990s was caused by an under-registration due to the lack of mandatory registration of myelodysplastic syndromes by cancer registries in those years. An increasing incidence trend was generally observed for all groups except for Chronic Lymphocytic Leukemia/Small Lymphocytic Lymphoma (CLL/SLL), which showed a decreasing trend in the last period.

**Table 4:** Reggio Emilia Cancer Registry. Distribution of hematological malignancies registered in the Province of Reggio Emilia by periods of diagnosis.

	1996-2000		2001-2005		2006-2010		2011-2015		2016-2020	
	n (%)	SR (95%CI)	n (%)	SR (95%CI)	n (%)	SR (95%CI)	n (%)	SR (95%CI)	n (%)	SR (95%CI)
Hodgkin Lymphoma (HL)	86 (17.5)	3.8 (3.0-4.6)	92 (18.7)	3.8 (2.9-4.6)	105 (21.3)	4.3 (3.5-5.1)	106 (21.5)	4.1 (3.3-4.9)	103 (20.9)	4.0 (3.2-4.8)
Chronic Lymphocytic Leukemia/Small Lymphocytic Lymphoma (CLL/SLL)	146 (16.5)	6.5 (5.5-7.6)	221 (25.0)	9.3 (8.1-10.6)	202 (22.9)	7.9 (6.8-9.1)	209 (23.7)	7.6 (6.6-8.6)	105 (11.9)	3.5 (2.8-4.2)
Lymphoplasmacytic Lymphoma (LPL)	33 (17.2)	1.4 (1.0-2.0)	35 (18.2)	1.5 (1.0-2.0)	38 (19.8)	1.5 (1.1-2.0)	37 (19.3)	1.4 (0.9-1.8)	49 (25.5)	1.6 (1.2-2.1)
Marginal Zone Lymphoma (MZL)	20 (5.9)	0.9 (0.5-1.3)	90 (26.4)	3.8 (3.0-4.6)	85 (24.9)	3.3 (2.6-4.0)	77 (22.6)	2.8 (2.2-3.4)	69 (20.2)	2.6 (1.9 (3.0)
Follicular Lymphoma (FL)	42 (10.1)	1.9 (1.3-2.5)	72 (17.3)	3.0 (2.3-3.8)	77 (18.5)	3.2 (2.4-3.9)	100 (24.0)	3.7 (3.0-4.4)	125 (30.0)	4.4 (3.6-5.2)
Diffuse Large B-Cell Lymphoma (DLBCL)	131 (14.0)	5.8 (4.8-6.8)	169 (18.1)	7.0 (6.0-8.1)	232 (24.8)	9.0 (7.9-10.2)	202 (21.6)	7.3 (6.3-8.4)	202 (21.6)	7.0 (6.1-8.0)
Mature T-cell and NK-cell neoplasms	41 (14.5)	1.8 (1.3-2.4)	40 (14.2)	1.7 (1.2-2.3)	68 (24.1)	2.7 (2.1-3.3)	71 (25.2)	2.6 (2.0-3.2)	62 (21.0)	2.2 (1.6-2.7)
Mantle Cell Lymphoma (MCL)	15 (14.3)	0.7 (0.3-1.0)	14 (13.3)	0.6 (0.3-0.9)	22 (20.9)	0.9 (0.5-1.3)	29 (27.6)	1.0 (0.7-1.4)	25 (23.8)	0.9 (0.5-1.2)
Plasma Cell Neoplasms (PCN)	172 (15.0)	7.7 (6.6-8.9)	235 (20.5)	9.8 (8.5-11.0)	230 (20.1)	8.9 (7.8-10.1)	262 (22.9)	9.5 (8.3-10.6)	247 (21.6)	8.5 (7.4-9.6)
Acute Lymphoblastic Lymphoma/leukemia (ALL)	35 (16.9)	1.9 (1.3-2.5)	38 (18.4)	1.8 (1.2-2.3)	38 (18.4)	1.5 (1.0-2.0)	46 (22.2)	1.8 (1.2-2.3)	50 (24.1)	1.9 (1.4-2.4)
Myeloproliferative Neoplasms (MPN)	91 (8.4)	4.0 (3.2-4.9)	195 (18.0)	8.1 (7.0-9.3)	236 (21.8)	9.4 (8.2-10.6)	306 (28.2)	11.0 (9.8-12.3)	256 (23.6)	9.0 (7.9-10.1)
Myelodysplastic Syndromes (MDS)	6 (0.8)	0.3 (0.1-0.5)	88 (12.4)	3.6 (2.8-4.3)	148 (20.9)	5.5 (4.6-6.4)	251 (35.4)	8.6 (7.5-9.6)	216 (30.7)	6.9 (6.0-7.9)
Acute Myeloid Leukemia (AML)	131 (19.6)	5.8 (4.8-6.8)	115 (17.2)	4.7 (3.8-5.5)	122 (18.2)	4.7 (3.9-5.6)	145 (21. 7)	5.3 (4.4-6.1)	156 (23.3)	5.2 (4.4-6.1)
Others	5 (4.3)	0.2 (0.0-0.4)	13 (11.2)	0.5(0.2-0.8)	52 (44.8)	2.0 (1.5-2.5)	25 (21.6)	0.9 (0.5-1.2)	125 (21.5)	
**Total**	**954 (12.6)**		**1,417 (18.7)**		**1,655 (21.8)**		**1,866 (24.6)**		**1,686 (22.2)**	

SR, age-Standardized Rates (European Standard Population 2013).

**Figure 1:**
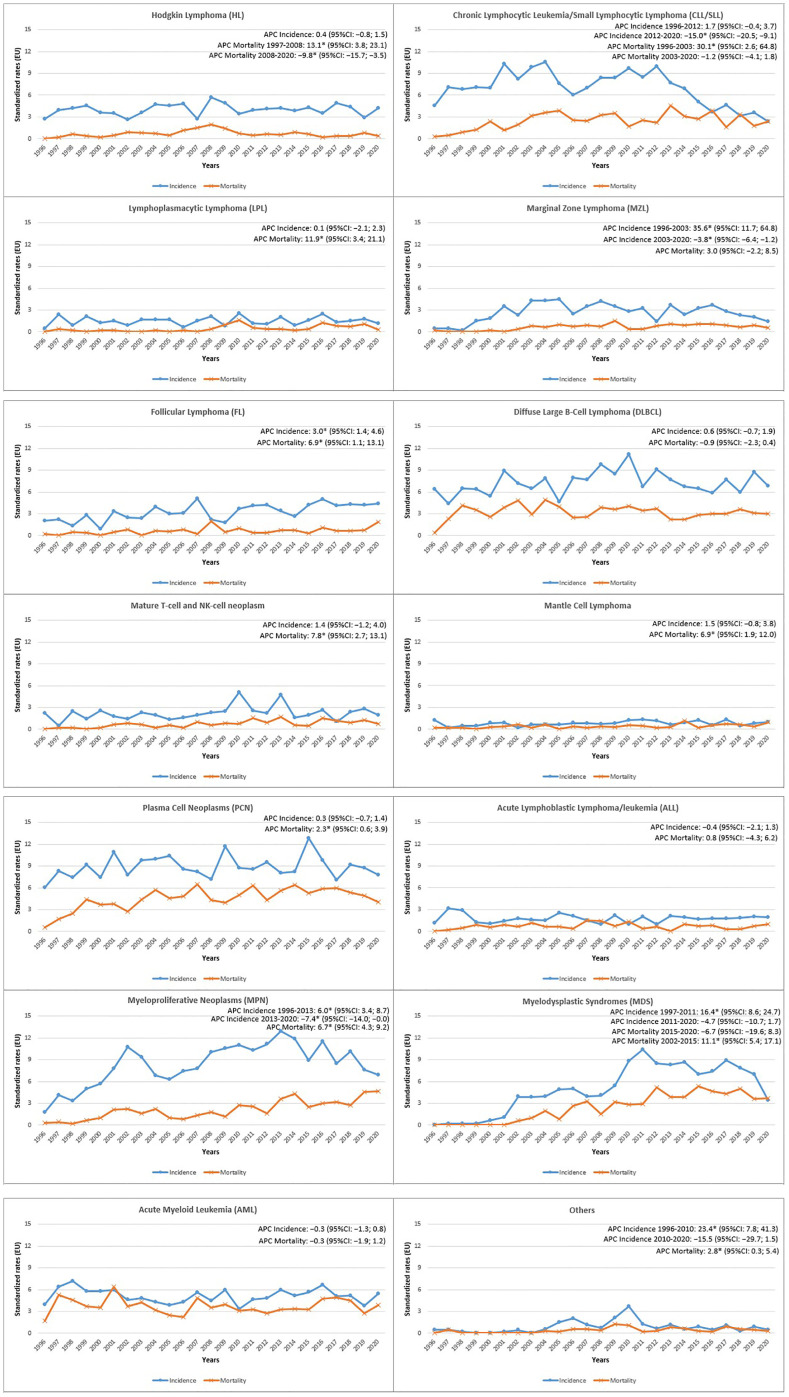
Reggio Emilia Cancer Registry. Incidence and mortality age-Standardized Rates (European Standard Population 2013) per 100,000 person-years (p-y) in the Province of Reggio Emilia in the period 1996-2020 by HMs categories.

Annual age-standardized incidence and mortality rates were used to describe HMs trends from 1996 to 2020 (**Figure 1**). A significant increase in the incidence rate was registered for FL (Annual Percent Change, APC: 3.0%; 95%CI 1.4, 4.6). On the contrary, MPN showed an initial increase until 2013 (APC: 6.0%; 95%CI 3.4, 8.7), followed by a significant decrease (APC: -7.4%; 95%CI -14.0, -0.0). An increasing trend was also observed for MDS but only in the first period 1997-2011 (APC: 16.4%; 95%CI 8.6, 24.7) and for the MZL only in the earlier years 1996-2003 (APC: 35.6%; 95%CI 11.7, 64.8), followed by a significant decrease in the subsequent years 2003-2020 (APC: -3.8; 95%CI -6.4,-1.2). For CLL/SLL incidence a decreasing trend was appreciable since 2012 (APC: −15.0%; 95%CI -20.5, -9.1).

The mortality trend showed a significant increase for HL until 2008 (APC: 13.1%; 95%CI 3.8, 23.1), followed by a substantial decrease in the last period (APC: -9.8%; 95%CI -15.7, -3.5) (**Figure 1**). The mortality also decreased for DLBCL (APC: -0.9%; 95%CI -2.3, 0.4), showing an increasing trend for Mature T-cell and NK-cell neoplasms (APC: 7.8%; 95%CI 2.7, 13.1). Furthermore, mortality increased for MPN (APC: 6.7%; 95%CI 4.3, 9.2), MDS only until 2015 (APC: 11.1%; 95%CI 5.4, 17.1), and increased for LPL in the entire period (APC: 11.9%; 95%CI 3.4, 21.1). CLL/SLL mortality, after a strong increase from 1996 to 2003 (APC: 30.1%; 95%CI 2.6, 64.8), was rather stable.

**Figure 2:**
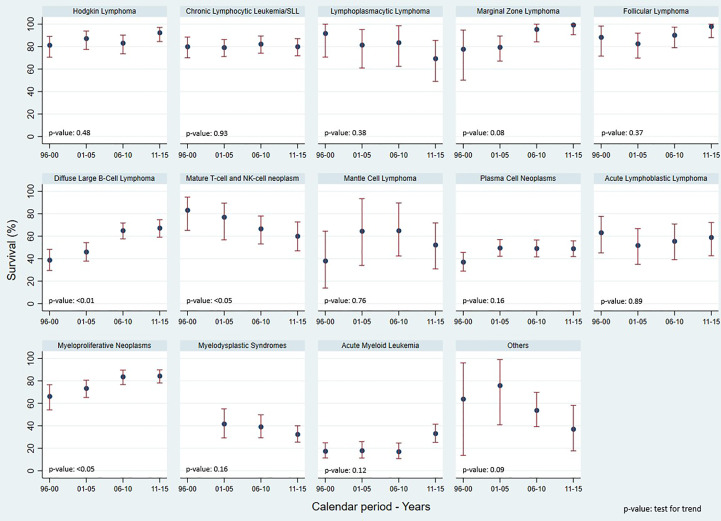
Reggio Emilia Cancer Registry. 5-yr Relative Survival, test for trends using Poisson models, by HMs categories and calendar period.

Concerning the 5-year Relative Survival (RS) (**Figure 2**), HL showed an increase from 81.3% in 1996-2000 to 92.4% in 2011-2015. The 5-year RS also increased for FL from 88.4% to 98.0%, DLBCL from 38.8% to 67.3%, Acute Myeloid Leukemia (AML) from 17.5% to 33.2%, and MPN from 66.3% to 84.4%. A decrease in 5-year RS was observed for Mature T-cell and NK-cell neoplasms, from 47.8% to 39.4%, for MDS from 41.8% to 32.5%, and for LPL from 91.8% to 69.4%.

**Table 5** compares incidence, mortality, and survival recorded in RE-CR with those recorded in the US Surveillance, Epidemiology, and End Results (SEER) program. In general, when comparable, the incidence rates recorded in our study are slightly higher than those in the US, except for CLL/SLL. The mortality data align with those from the US registry, whereas survival shows higher values except for CLL/SLL and PCN.

**Table 5:** Incidence and mortality age-standardized rates, and 5-year survival rates of the hematological malignancies: comparison between the Reggio Emilia Cancer registry and the US SEER (Surveillance, Epidemiology and End Results) data.

	Incidence		Mortality		5-year Relative Survival	
	RE-CR	SEER	RE-CR	SEER	RE-CR	SEER
	SR (95%CI)	SR	SR(95%CI)	SR	% (95%CI)	%
Hodgkin Lymphoma (HL)	4.2 (2.4-6.0)	2.4	0.4 (0.0-1.0)	0.2	92 (84-97)	89
Chronic Lymphocytic Leukemia/Small Lymphocytic Lymphoma (CLL/SLL)	2.4 (1.2-3.6)	3.8	2.4 (1.1-3.7)		80 (72-87)	88
Lymphoplasmacytic Lymphoma (LPL)	1.2 (0.3-2.0)		0.3 (0.0-0.8)		69 (49-85)	
Marginal Zone Lymphoma (MZL)	1.4 (0.5-2.4)		0.5 (0.0-1.2)		99 (91-100)	
Follicular Lymphoma (FL)	4.4 (2.7-6.1)	2.3	1.8 (0.7-3.0)	0.4	98 (88-100)	90
Diffuse Large B-Cell Lymphoma (DLBCL)	6.8 (4.6-9.2)	5.3	3.0 (1.6-4.4)	1.5	67 (59-75)	65
Mature T-cell and NK-cell neoplasms	1.9 (0.8-3.1)		0.7 (0.1-1.5)		59 (47-73)	
Mantle Cell Lymphoma (MCL)	1.0 (0.2-1.8)		0.9 (0.2-1.7)		52 (31-72)	
Plasma Cell Neoplasms (PCN)	7.8 (5.6-10.0)	7.1	4.1 (2.4-5.7)	3.1	49 (42-56)	58
Acute Lymphoblastic Lymphoma/leukemia (ALL)	1.9 (0.7-3.1)	1.7	0.8 (0.2-1.7)	0.4	59 (42-72)	65
Myeloproliferative Neoplasms (MPN)	6.9 (4.7-9.0)		4.7 (2.9-6.4)		84 (78-90)	
Myelodysplastic Syndromes (MDS)	3.4 (2.0-4.9)		3.7 (2.2-5.2)		32 (26-40)	
Acute Myeloid Leukemia (AML)	5.5 (3.6-7.3)	4.1	3.9 (2.3-5.4)	2.6	33 (25-41)	31

RE-CR, Reggio Emilia Cancer Registry; SR, age-Standardized Rates

Regarding RE-CR the following data are presented: Incidence 2020, Mortality 2020, Survival 2011-2015.

Regarding SEER the following data are presented: Incidence estimate 2022, Mortality estimate 2022, Survival 2012-2018. https://seer.cancer.gov/statfacts/html/hodg.html, consulted on 16-02-2023.

## 4 Discussion

HMs represent a heterogeneous group of neoplasms with many challenges in the diagnostic pathway. Our work aimed to describe HMs’ behaviors over 25 years of registration by RE-CR from 1996 to 2020 through new dedicated software that automatically identified suspected HMs cases by combining several databases.

It is an accepted notion that the consultation of the three historical archives (anatomy, hospital discharge records, and mortality) is sufficient for solid tumors CRs, in which two variables (topography and morphology) are mandatory (27). Concerning HMs, we have previously shown that including additional information sources improves the completeness of cases by 4.2% in these neoplasms (17). The use of our algorithm allowed us to enhance the collection, coding, and classification quality of HM registration. Artificial Intelligence (AI) applications should facilitate this approach in terms of timeliness and sensitivity (28–30). Learning algorithms are, for example, applicable to produce stochastic estimators (e.g. positive predictive value for the HM) and could increase the automatism of our current process. Attempts have been made in our Institute to use AI for diagnostics (31–34), the treatment of some neoplasms (35–37), or screening programs (38–40). Even if the present study is limited to only one Italian CR, it may contribute to a proper CR classification for hematologists in their clinical practice.

Our study reports 7,578 cases of HMs; they affected mainly males (except for Follicular Lymphoma) and elderly patients with a median age of 70 years except for HL (median age of 40 years), and ALL (median age of 21 years).

Concerning single malignancies, HL incidence rates registered in our study (3.9 and 4.5 cases x 100,000, respectively, in males and females) are very similar to those reported in Northern Italy (4.1 and 3.4 cases per 100.000 in males and females, respectively) (18) and close to data from other western countries (41). Five-year RS has increased compared to the early study periods (92.4% in 2011-2015 *vs.* 81.3% in 1996-2000), with similar findings to a previous Italian study (42), confirming the high curability of HL. Our data did not confirm the higher mortality rates reported in a recent population-based study (Excess Mortality Ratio = 1.26; 95% CI 1.04-1.54) (11).

For NHL, the US and Italian data report a stable incidence trend in recent years with a significant drop in mortality and increase in survival, which is mainly observed for B cell NHL because of the introduction of immunotherapy in the late ‘90s (3, 43–48). Our study described incidence, mortality, and survival for each primary NHL subtype. With some limitations due to the small number of rarer histotypes, we showed a significant drop in the incidence rates of CLL/SLL and MZL and an increase in FL. All other NHL subtypes had stable rates. Mortality rates had similar trends compared to incidence, despite survival increased during the study period for B-cell Lymphomas, achieving statistical significance for DLBCL patients. Similar to our results a Swedish study reported a 47% increase in the 5-year prevalence of NHL overall in 2016 compared to 2004: these results shall be used to better evaluate the burden of disease and to improve healthcare planning and resource allocation (12). The observation of a notable improvement in DBCL survival is an expected finding that is mainly due to the introduction of antiCD20 monoclonal antibodies in addition to the polychemotherapy treatment since the early 2000s (49) and might also have been the consequence of healthcare improvements, including increasing access to effective treatments for elderly patients (50).Of note, consistently due to their indolent nature and to the availability of treatments, the 5-year RS was close to 100% in both MZL and FL. Conversely, peripheral T-cell lymphoma (PTCL) was confirmed as the lymphoma subtype with relatively worse outcome reflecting the lack of effective therapies.

The incidence of PCN reported in our study is in line with that reported in northern Italy, with 11.2 and 8.1 cases per 100,000 per year, in males and females, respectively (18). Interestingly, the incidence decreased in both sexes while mortality was stable. The 5-year RS in Italy is 53%, slightly better than the 49% recorded in our analysis. Mortality also decreased (2); in particular, the introduction of proteasome inhibitors in 2003 and immunomodulators in 2006 led to a net increase in survival. For example, the increase of 5-year RS from 37% to 48% comparing cases diagnosed from 1994-2000, with those diagnosed between 2001-2006, was similar to the increase observed at the Mayo Clinic in the same periods (from about 35% to 45%) (7).

Concerning myeloid malignancies, they are generally more frequent in Western countries, with predominance in the male sex (6, 51), probably related to different exposures to risk factors and occupational exposures (52) and aging (13). However, since the group includes a considerable variety of subtypes, incidence and survival can present different values even within the same category: for example, the 5-year survival of AML varies from 5% for aggressive entities like therapy-related AML to 85% for indolent/treatable conditions like chronic myeloid leukemia; again males usually experience worse survivals than females (48.8% *vs.* 60.4%, respectively) (13).

The completeness and the accuracy of the clinical and pathological information is a strength of our study. Also, ours is the only Cancer Registry in Italy that explored what number of additional cases could be detected through accessing information sources other than those routinely feeding Cancer Registries. Our results suggest that such procedure would be important, as well-informed population studies are required to inform aetiological hypothesis, healthcare planning, and to assess the impact of new therapies (17).. Furthermore, it is a population-based study, so there is not only information from hospital centres or patients enrolled in clinical trials, and today more than ever we need population-based longitudinal data to inform aetiological hypothesis, healthcare planning, and impact assessment of the introduction of new therapies (13, 14).

The lack of individual information, on treatments that substantially impacted survival in recent years and comorbidities, which can influence the choice of treatments and, therefore, the outcome limits the interpretation of our results. Moreover, some of the categories that were clinically based and may not fully correspond to conventional classifications, Finally, the small numbers observed in some predefined groups made it impossible to analyze them separately and we had to merge them with pathologically close groups. These choices might affect the precision of some of our observations but wouldn’t affect the study’s main message.

In conclusion, we provided updated about HM incidence and outcomes based on the Cancer Registry of Reggio Emilia province, a highly productive and industrialized territory representative of Northern Italy. More importantly, our study could provide the basis for future programs on HM control, patient care, and cancer research programs. This goal can be reached by offering, for the future, well-structured databases including biomolecular information as suggested by the WHO classification to allow also the cross-validation of new prognostic indicators for HM.

## Data availability statement

The raw data supporting the conclusions of this article will be made available by the corresponding author, without undue reservation.

## Ethics statement

This population-based cohort study uses data from the Reggio Emilia Cancer Registry, approved by the Provincial Ethics Committee of Reggio Emilia (ref. no. 2014/0019740 of 4 August 2014). The Ethics Committee authorized, even in the absence of consent, the processing of personal data, including those suitable for revealing the state of health of patients who are deceased or untraceable for the execution of the study.

## Author contributions

Conceptualization, investigation, writing—original draft, visualization, and supervision: LM. Conceptualization, investigation, and writing—original draft: DP. Formal analysis, visualization, and supervision: FMa. Investigation, supervision, and visualization: FR. Writing—review and editing, and visualization: IB. Supervision: FMo. Supervision: AR. Supervision: BG. Supervision: AT. Supervision: RV. Supervision: LA. Supervision: MI. Visualization and supervision: PG. Visualization and supervision: CV. Visualization and supervision: FM. Visualization and supervision: AN. Conceptualization, writing—original draft, investigation, and supervision: SL. All authors have read and agreed to the published version of the manuscript. All authors contributed to the article.
