# Supplementary material for: Incidence, mortality, and survival of hematological malignancies in Northern Italian patients: an update to 2020
Source: Front Oncol. 2023 Jul 18;13:1182971. doi: 10.3389/fonc.2023.1182971 (PMC10391155; doi:10.3389/fonc.2023.1182971)
Supplement: Supplementary file 1 [file Table_1.docx]

|  | Male | | Female | |
| --- | --- | --- | --- | --- |
|  | **n.** | **DSR** | **n** | **DSR** |
| Hodgkin Lymphoma (HL) | 11 | 4.6 | 11 | 3.8 |
| Chronic Lymphocytic Leukemia/Small Lymphocytic Lymphoma (CLL/SLL) | 7 | 2.6 | 8 | 2.2 |
| Lymphoplasmacytic Lymphoma (LPL) | 2 | 0.7 | 5 | 1.6 |
| Marginal Zone Lymphoma (MZL) | 6 | 1.0 | 3 | 2.0 |
| Follicular Lymphoma (FL) | 9 | 3.4 | 17 | 5.1 |
| Diffuse Large B-Cell Lymphoma (DLBCL) | 21 | 8.0 | 17 | 5.8 |
| Mature T-cell and NK-cell neoplasms (not cutaneous) | 1 | 0.3 | 4 | 1.4 |
| Plasma Cell Neoplasms (PCN) | 23 | 8.5 | 25 | 7.3 |
| Myeloproliferative Neoplasms (MPN) | 23 | 8.5 | 17 | 5.5 |
| Myelodysplastic Syndromes (MDS) | 12 | 4.2 | 11 | 3.1 |
| Acute Myeloid Leukemia (AML) | 14 | 5.1 | 20 | 6.0 |
| Others | 16 | 6.1 | 9 | 3.0 |

Supplementary table. Age standardized Incidence rates. Reggio Emilia, year 2020, by sex.

DSR= Directly age-Standardized Rates
